# Supplementary material for: Second-Generation Phage Lambda Platform Employing SARS-CoV-2 Fusion Proteins as a Vaccine Candidate
Source: Vaccines (Basel). 2024 Oct 22;12(11):1201. doi: 10.3390/vaccines12111201 (PMC11598875; doi:10.3390/vaccines12111201)
Supplement: Supplementary file 1 [file vaccines-12-01201-s001.zip › vaccines-3240706-supplementary.pdf]

vaccines

MDPI

Article | Supplementary Material

Second-Generation Phage Lambda Platform Employing SARS-CoV-2 Fusion Proteins as a Vaccine Candidate

Alexis Catala [1,†], Bennett J. Davenport [2,†], Thomas E. Morrison [2] and Carlos E. Catalano [1,*]

1 Department of Pharmaceutical Sciences, Skaggs School of Pharmacy and Pharmaceutical Sciences, University of Colorado Anschutz Medical Campus, Aurora, CO 80045, USA; alexis.catala@cuanschutz.edu
2 Department of Immunology and Microbiology, University of Colorado Anschutz Medical Campus, Aurora, CO 80045, USA; bennett.davenport@cuanschutz.edu (B.J.D.); thomas.morrison@cuanschutz.edu (T.E.M.)
* Correspondence: carlos.catalano@cuanschutz.edu; Tel.: +1-303-724-0011
† These authors contributed equally to this work.

Content: Figures

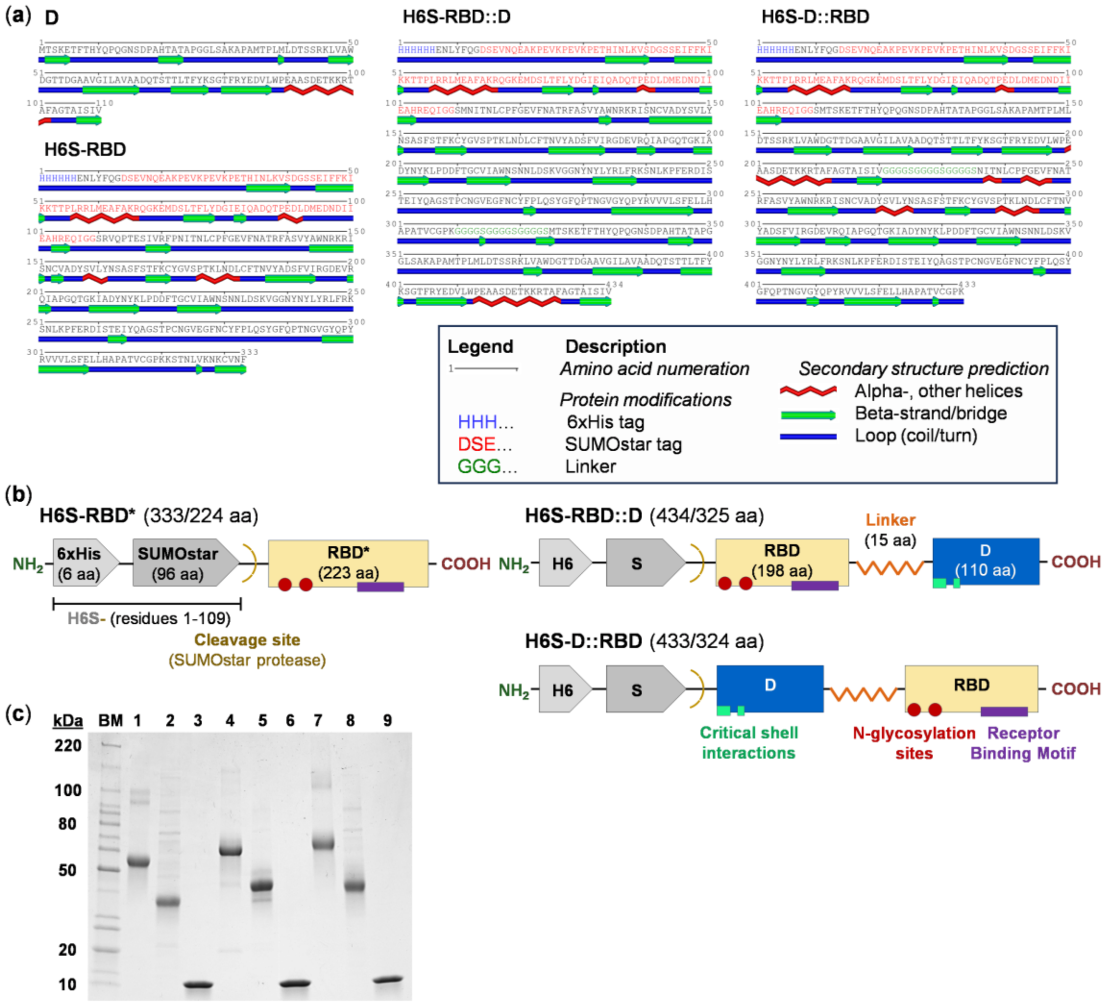

Figure S1. Protein design and purification. (**a**) Secondary structure prediction analysis (SABLE server, https://sable.cchmc.org/). (**b**) Schematic of the RBD constructs. (**c**) Purified proteins analyzed by sodium dodecyl-sulfate polyacrylamide gel electrophoresis (SDS-PAGE) following Coomassie Blue staining. Lanes: BM, BenchMark Ladder (10-220 kDa); 1, H6S-RBD (36.1 kDa); 2, RBD (23.4 kDa); 3, 6 and 9, D (11.6 kDa); 4, H6S-RBD::D (47.5 kDa); 5, RBD::D (34.9 kDa); 7, H6S-D::RBD (47.4 kDa); 8, D::RBD (34.8 kDa). Molecular weights provided are based on protein amino acid sequence. The N-

terminal fusion tag (H6S-) causes a mass shift of ~17 kDa, resulting in larger apparent masses for the tagged RBD constructs, and protein glycosylation will also cause a mass shift. Abbreviations: SARS-CoV-2, severe acute respiratory syndrome coronavirus 2; lambda, bacteriophage lambda; RBD, receptor-binding domain in the S1 fragment of the SARS-CoV-2 spike glycoprotein; D, lambda decoration protein (gpD); H6S-, fusion tag consisting of hexahistidine (6xHis, H6) and SUMOstar (S) tags. *3.2.Engineering the RBD Constructs*

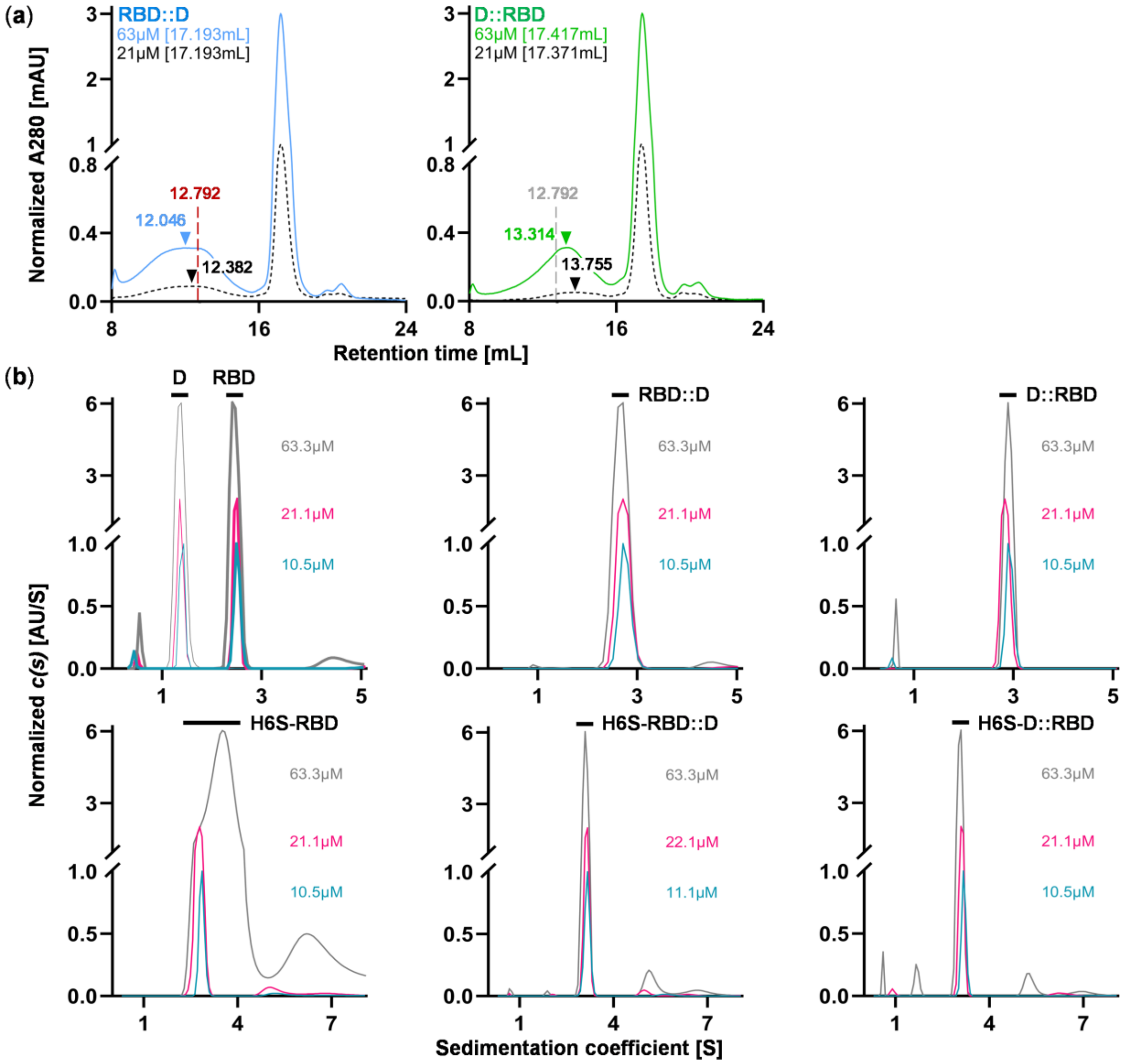

Figure S2. Protein biophysical characterization. (**a**) Overlay of SEC chromatograms for the RBD fusions at 21 µM (black, short-dashed line) and 63 µM (solid line – blue, RBD::D; green, D::RBD). Traces represent an average of three independent injections at either concentration, and the signal (A280, absorbance at 280 nm) was normalized to 1 and adjusted using a correction factor relative to 21 µM. As a reference for the high-molecular weight species observed in the RBD fusion samples, the elution volume of thyroglobulin (calibration standard, 669 kDa) is depicted as a red or grey long-dashed line in the RBD fusion plots. (**b**) Sedimentation velocity analytical ultracentrifugation (SV-AUC) studies to characterize protein self-association and oligomeric state. Collectively, the plots provide an overlay of the sedimentation coefficient distributions of the proteins (untagged, *top*; tagged, *bottom*) at three concentrations: cyan, 10.5/11.1 µM (0.1-0.5 mg/mL); magenta, 21.1/22.1 µM (0.2-1.1 mg/mL); grey, 63.3 µM (0.7-3.0 mg/mL). Absorbance data (250, 280 or 295 nm) was acquired at 20 °C with a rotor speed of 50k rpm, and the best-fit *c(s)* distributions were normalized (scale, 0-1) and adjusted for concentration. *3.3. RBD Construct Characterization*

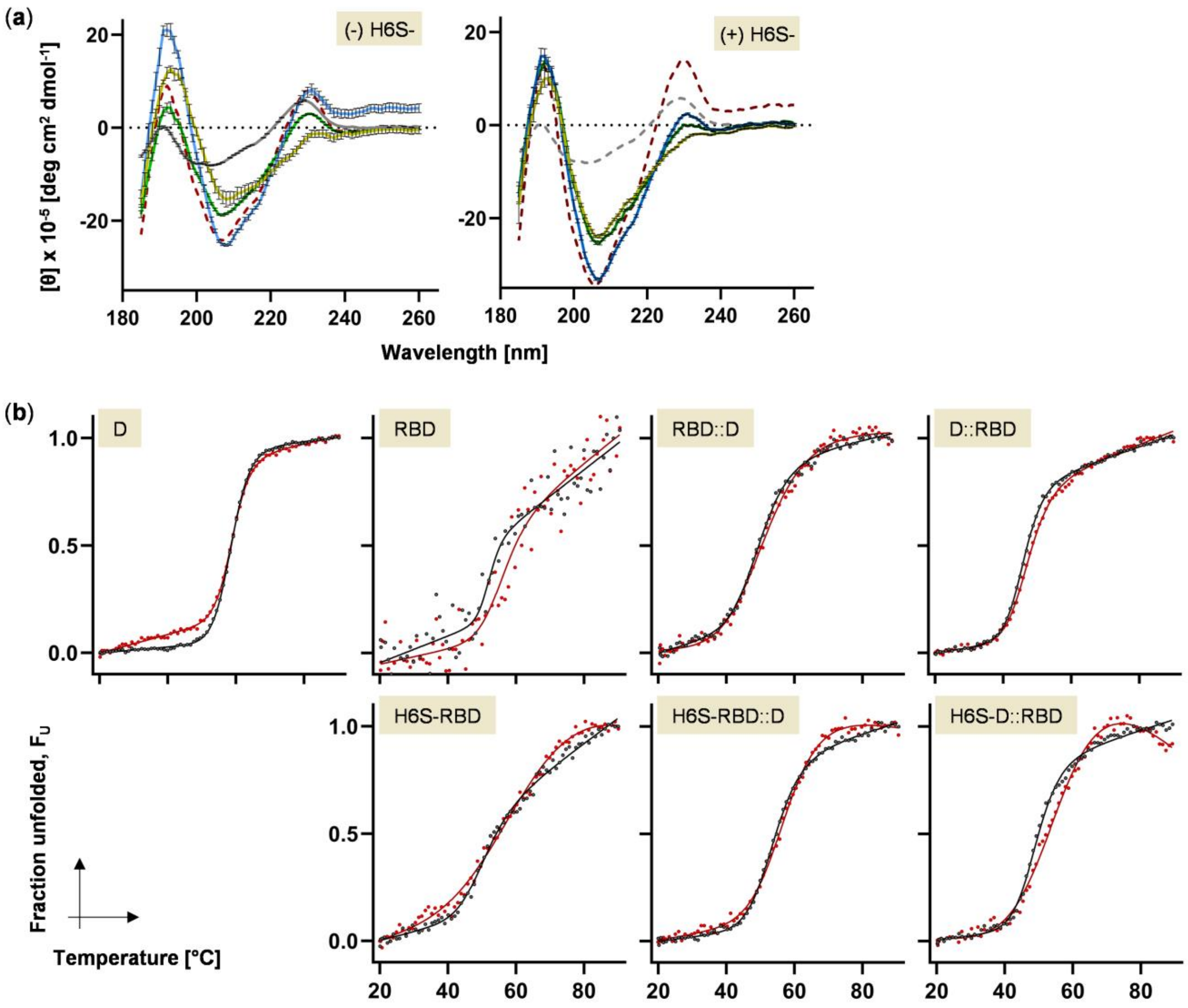

Figure S3. Assessment of protein secondary structure and thermal stability. Purified proteins in 10 mM sodium phosphate [pH 6.7] buffer were analyzed by circular dichroism (CD) spectroscopy. (**a**) Far-UV spectra overlay for the untagged (*left*) and tagged (*right*) proteins in standard units of molar ellipticity (mean ± error, 8 replicate scans). Proteins (12-48 kDa): grey, D (35 µM, 0.4 mg/mL); yellow, RBD (4 µM, 0.1 mg/mL); dark yellow, H6S-RBD (8 µM, 0.3 mg/mL); blue, RBD::D (6 µM, 0.2 mg/mL); dark blue, H6S-RBD::D (6 µM, 0.3 mg/mL); green, D::RBD (12 µM, 0.4 mg/mL); dark green, H6S-D::RBD (8 µM, 0.4 mg/mL). The theoretical spectra for the RBD fusions is shown as a red dashed line. (**b**) Thermal melting curves at 230 nm (black) and 222 nm (red). Refer to Table S5 for the calculated transition midpoint values. *3.3. RBD Construct Characterization*

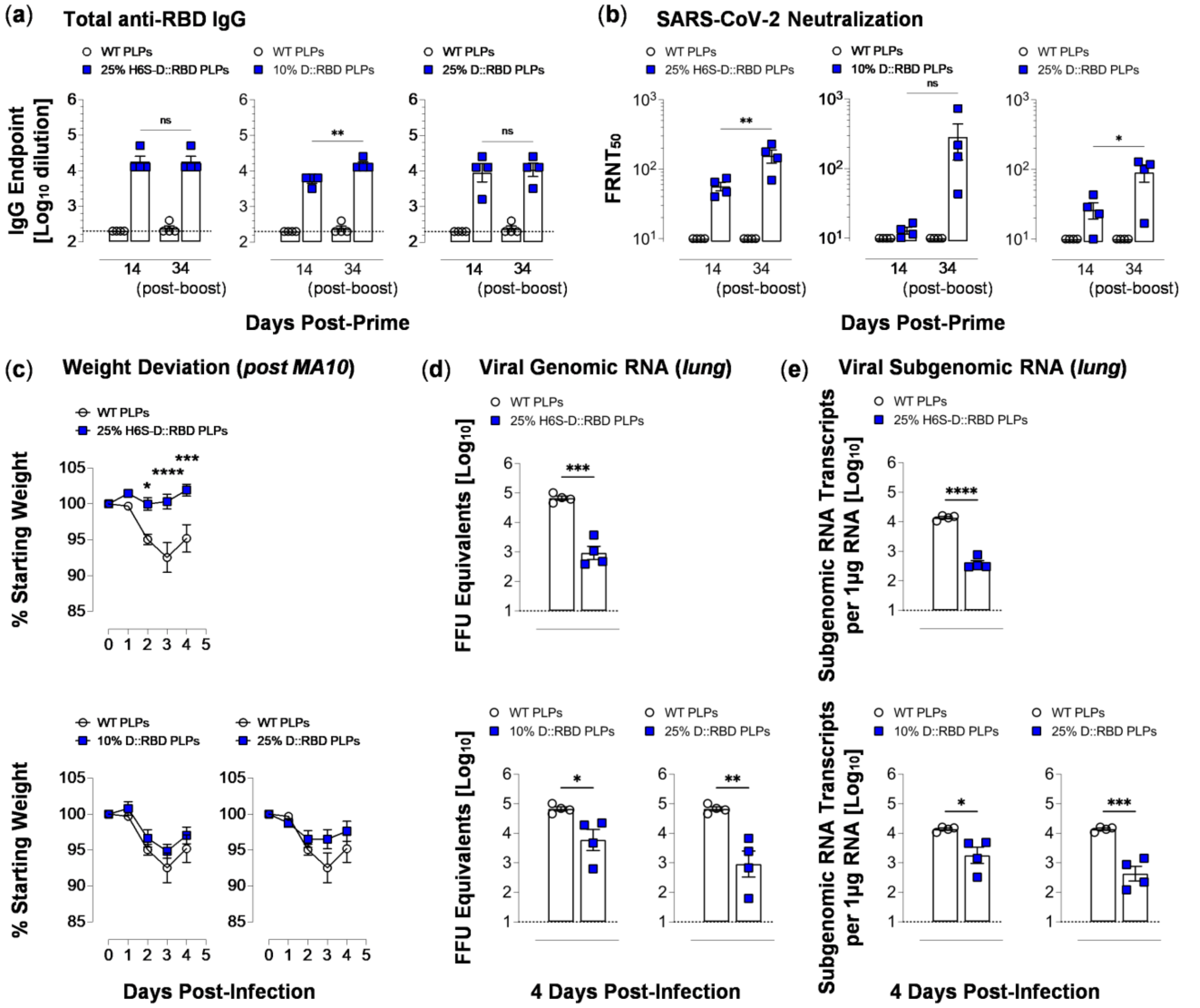

Figure S4. Immunization using PLPs decorated with 25% H6S-D::RBD, 10% D::RBD and 25% D::RBD confers protection against SARS-CoV-2 infection. BALB/c mice were immunized with 2.5 µg of WT PLPs (control; open circles) or PLPs decorated with 25% H6S-D::RBD or D::RBD at either 10% or 25% surface density (blue squares) *via* intramuscular inoculation (n = 4 mice/group). At 21 days post-prime, mice received a boost at the same dose and route. (**a**,**b**) Serum samples collected at 14 and 34 days post-primary (13 days post-boost) immunization were analyzed for total anti-RBD IgG by ELISA (**a**) and neutralizing activity against live SARS-CoV-2 by a focus-reduction neutralization test (FRNT) (**b**). Error bars represent the mean ± SEM. *P*-values determined by one-way ANOVA with Tukey's multiple comparisons test: $*p < 0.05$, $**p < 0.01$. (**c**) At 35 days post-prime (14 days post-boost), mice were challenged intranasally with 10^4 PFU of SARS-CoV-2, strain MA10 and weight was monitored daily. Error bars represent the mean ± SEM. *P*-values were determined by two-way ANOVA with Sidak's multiple comparisons test: $*p < 0.05$, $***p < 0.001$, $****p < 0.0001$. (**d**,**e**) At 4 days post-infection, the viral burden in lung tissue was quantified by RT-qPCR for viral genomic RNA (**d**) and N subgenomic RNA (**e**). *P*-values were determined by unpaired students t-test: $*p < 0.05$, $**p < 0.01$, $***p < 0.001$, $****p < 0.0001$. *3.5. H6S-D::RBD PLPs are Immunogenic, 3.6. Vaccination with H6S-D::RBD PLPs Protects Against Virulent SARS-CoV-2 Challenge*

Content: Tables

Table S1. Secondary structure prediction analysis

Protein	SABLE II[1] (predicted structure, %)[2]		
	H	E	C
D	12	36	52
RBD	5	29	66
RBD::D	4	32	63
D::RBD	10	28	61
H6S-RBD	8	27	65
H6S-RBD::D	6	30	64
H6S-D::RBD	11	27	62

[1] Sequence-based prediction performed using the SABLE server, version SABLE II

[2] Code: H, α- and other helices; E, β-strand/bridge/sheet; C, coil (loop/turn)

Table S2. Biophysical characterization using size exclusion chromatography

Protein	M_{aa}	SEC Analysis[1]			
		V_e	K_D	M_r	R_S[2]
D	11572	18.397 ± 0.15	0.70 ± 0.02	21663 ± 180	2.15 ± 0.10
RBD	25185	17.864 ± 0.04	0.63 ± 0.01	31423 ± 861	2.54 ± 0.03
RBD::D	34929	17.193 ± 0.00	0.55 ± 0.00	50472 ± 3692	3.13 ± 0.00
D::RBD	34798	17.383 ± 0.06	0.57 ± 0.01	44145 ± 2661	2.95 ± 0.05

Abbreviations: SEC, size exclusion chromatography; M_{aa}, molecular weight based on amino acid (aa) composition [Da]; V_e, elution volume [mL]; K_D, partition/distribution coefficient; M_r, relative molecular mass based on a hydrated state [Da]; R_S, Stokes radius [nm]; SD, standard deviation

[1] Values provided are an average of three independent injections, reported as mean +/- SD

[2] R_S values of proteins used as calibration standards with a molecular weight similar to study proteins (molecular shape, globular) [nm]: 1.70, cytochrome c (12 kDa); 2.20, trypsin inhibitor (22 kDa); 2.75, β-lactoglobulin (35 kDa)

Table S3. Fit parameters derived from the sedimentation velocity studies

Protein	M_{aa}	SV-AUC Analysis					
		Conc	λ	s	$s_{20,W}$	f/f_0	M_f
D	11.6	10.5 µM (0.12 mg/mL)	280	1.40	1.46	1.23	10.9
		21.1 µM (0.24 mg/mL)	280	1.37	1.42	1.33	11.8
		63.3 µM (0.73 mg/mL)	280	1.36	1.41	1.34	11.9
RBD	25.2	10.5 µM (0.26 mg/mL)	280	2.51	2.61	1.23	26.1
		21.1 µM (0.53 mg/mL)	280	2.49	2.59	1.21	25.2
		63.3 µM (1.59 mg/mL)	280	2.45	2.55	1.23	25.1
RBD::D	34.9	10.5 µM (0.37 mg/mL)	280	2.76	2.86	1.40	35.7
		21.1 µM (0.74 mg/mL)	280	2.72	2.82	1.46	37.3
		63.3 µM (2.21 mg/mL)	280	2.67	2.77	1.52	38.5
D::RBD	34.8	10.5 µM (0.37 mg/mL)	280	2.93	3.04	1.23	32.0
		21.1 µM (0.73 mg/mL)	280	2.84	2.95	1.36	35.0
		63.3 µM (2.20 mg/mL)	280	2.90	3.01	1.21	30.7
H6S-RBD	37.8	10.5 µM (0.40 mg/mL)	280	2.86	2.97	1.21	30.7
		21.1 µM (0.80 mg/mL)	280	2.77	2.88	1.27	31.6
		63.3 µM (2.39 mg/mL)	280	3.44	3.57	1.18	50.4
H6S-RBD::D	47.5	11.1 µM (0.53 mg/mL)	280	3.16	3.28	1.45	46.3
		22.1 µM (1.05 mg/mL)	280	3.14	3.26	1.47	46.8
		63.3 µM (3.01 mg/mL)	295	3.10	3.22	1.40	42.7
H6S-D::RBD	47.4	10.5 µM (0.50 mg/mL)	250	3.18	3.30	1.17	33.8
		21.1 µM (1.00 mg/mL)	250	3.11	3.23	1.22	34.8
		63.3 µM (3.00 mg/mL)	250	3.06	3.18	1.40	41.7

Abbreviations: SV-AUC, sedimentation velocity analytical ultracentrifugation; M_{aa}, molecular weight based on aa composition [kDa]; Conc, concentration [µM, mg/mL]; λ, wavelength [nm]; s, sedimentation coefficient [S]; $s_{20,w}$, sedimentation coefficient transformed to standard conditions of water at 20 °C [S]; f/f_0, frictional ratio (shape factor); M_f, molar mass based on frictional ratio (scale relationship) [kDa]

Table S4. Secondary structure analysis using circular dichroism spectroscopy

Protein	BeStSel (SS content, %)[1]				DichroWeb (SS fraction, %)[2]			
	H (1:2)	S (A1/2/3:P)	T	O	H (1:2)	S (1:2)	T	U
D	2.2 (0:2)	40.5 (0/17/24:0)	9.7	47.6	2 (1:1)	36 (21:15)	27	35
RBD	10.3 (3:7)	32.6 (0/15/12:6)	10.2	46.9	6 (2:4)	37 (24:13)	23	34
RBD::D	7.7 (5:3)	35.4 (0/14/22:0)	10.1	46.8	4 (1:3)	40 (26:14)	23	33
D::RBD	9.1 (4:5)	32.2 (0/16/16:0)	12.4	46.3	3 (0:3)	40 (26:14)	25	32
H6S-RBD	12.3 (6:6)	26.8 (0/13/12:3)	12.4	48.5	6 (1:5)	36 (23:13)	25	33
H6S-RBD::D	9.7 (4:5)	31.0 (0/15/17:0)	12.5	46.8	4 (1:3)	38 (24:14)	25	33
H6S-D::RBD	8.6 (5:4)	33.2 (1/16/17:0)	12.9	45.3	4 (1:3)	40 (26:14)	22	34

[1] BeStSel server (https://bestsel.elte.hu/information.php); NRMSD: 0.015-0.034; code: H, α-helix - regular (1), distorted (2); S, β-sheet/stand – antiparallel (A1, left-twisted/2, relaxed/3, right-twisted), parallel (P); T, turn; O, other (3,10-helix, π-helix, β-bridge, bend, loop/irregular region of structure)

[2] DichroWeb server (http://dichroweb.cryst.bbk.ac.uk/html/home.shtml); algorithm, reference set: CDSSTR, Set 3; NRMSD: 0.031-0.065; code: H, α- and 3,10-helices - regular (1), distorted (2); S, β-sheet/stand – regular (1), distorted (2); T, turn; U, unordered (one residue long turn/bend, π-helix, β-bridge, loop/irregular region of structure)

Table S5. Nonlinear regression analysis to assess protein stability

Protein	Wavelength, 230 nm				Wavelength, 222 nm				T_M (avg)
	T_M	ΔT_M (D)	ΔT_M (RBD)	ΔT_M (H6S-)	T_M	ΔT_M (D)	ΔT_M (RBD)	ΔT_M (H6S-)	
D	58.6 ± 0.05	-	6.74	-	59.0 ± 0.08	-	3.71	-	58.8 ± 0.25
RBD	51.9 ± 1.10	-6.74	-	2.21	55.2 ± 2.04	-3.71	-	-11.4	53.6 ± 2.39
RBD::D	49.8 ± 0.32	-8.77	-2.03	-3.81	51.6 ± 0.74	-7.31	-3.60	-6.14	50.7 ± 1.28
D::RBD	45.8 ± 0.09	-12.77	-6.03	-3.12	46.9 ± 0.19	-12.0	-8.31	-10.8	46.4 ± 0.78
H6S-RBD	49.6 ± 0.62	-8.95	-2.21	-	66.7 ± 4.54	7.69	11.4	-	58.2 ± 12.0
H6S-RBD::D	53.6 ± 0.17	-4.96	1.78	-	57.8 ± 0.38	-1.17	2.54	-	55.7 ± 2.93
H6S-D::RBD	49.0 ± 0.23	-9.65	-2.91	-	57.8 ± 0.75	-1.19	2.53	-	53.4 ± 6.23

Abbreviations: T_M, transition midpoint temperature [°C]

Table S6. Particle characterization using light scattering

Sample[1]	Zetasizer (solution)					
	**Z-Ave**	**PDI**	**I-PSD**	**V-PSD**	**N-PSD**	**ZP**
Naked PLPs	62.6 ± 0.38	0.06 ± 0.01	66.9 ± 0.63	56.4 ± 0.18	48.4 ± 0.16	-28.5 ± 0.61
WT PLPs	74.2 ± 1.02	0.13 ± 0.03	80.5 ± 0.38	63.6 ± 0.34	51.4 ± 0.51	-23.3 ± 1.82
10% RBD::D PLPs	106.7 ± 2.34	0.25 ± 0.02	123.4 ± 6.93	85.2 ± 1.80	54.5 ± 3.06	-16.0 ± 2.12
25% RBD::D PLPs	109.1 ± 0.81	0.20 ± 0.01	127.7 ± 3.96	93.6 ± 2.31	60.8 ± 3.60	-25.3 ± 1.70
50% RBD::D PLPs	106.5 ± 6.15	0.24 ± 0.06	129.9 ± 3.80	88.2 ± 5.73	52.5 ± 5.83	-21.2 ± 1.92
10% D::RBD PLPs	116.8 ± 3.13	0.25 ± 0.01	144.2 ± 10.5	96.9 ± 7.71	50.5 ± 8.03	-14.0 ± 1.27
25% D::RBD PLPs	88.8 ± 1.11	0.25 ± 0.01	102.6 ± 3.79	67.2 ± 1.74	46.0 ± 3.45	-26.8 ± 2.05
50% D::RBD PLPs	112.6 ± 25.7	0.41 ± 0.06	113.3 ± 26.5	66.8 ± 4.08	47.2 ± 3.92	-19.2 ± 0.71

Abbreviations: PLPs, phage-like particles; Z-Ave, weighted mean hydrodynamic size [d.nm]; PDI, polydispersity index; I-/V-/N-PSD, intensity/volume/number particle size distribution [d.nm]; ZP, zeta potential (overall surface charge) [mV]

[1]Naked PLPs are particles devoid of decoration protein (shell only); WT PLPs are wildtype particles decorated entirely with D (no foreign protein on shell); RBD fusion PLPs are particles decorated with chimeric protein at the indicated surface density percentage (remaining D-binding sites (420 total) were filled using D)
